# Research progress on pharmacological properties and application of probiotics in the fermentation of *Scutellaria baicalensis* Georgi

**DOI:** 10.3389/fnut.2024.1407182

**Published:** 2024-06-06

**Authors:** Fangyu Guo, Chunhai Li, Jiaxin Dou, Jie Liang, Zouquan Chen, Zhenshang Xu, Ting Wang

**Affiliations:** ^1^State Key Laboratory of Biobased Material and Green Papermaking, Qilu University of Technology, Shandong Academy of Science, Jinan, China; ^2^School of Bioengineering, Qilu University of Technology, Shandong Academy of Science, Jinan, China; ^3^Department of Radiology, Qilu Hospital, Cheeloo College of Medicine, Shandong University, Jinan, China

**Keywords:** *Scutellaria baicalensis Georgi*, probiotics, fermentation, application, pharmacological properties

## Abstract

*Scutellaria baicalensis* Georgi is a medicinal herb with a rich history of use in traditional Chinese medicine. This review concentrates on the chemical constituents of *Scutellaria baicalensis* Georgi, with a particular emphasis on flavonoids such as baicalin, baicalein, and wogonin. Additionally, it examines the effects of probiotic fermentation on the plant’s chemical profile and pharmacological actions. Evidence suggests that probiotic fermentation markedly modifies the bioactive components of *Scutellaria baicalensis* Georgi, thereby augmenting its medicinal potency. The paper delves into the mechanisms by which the primary active constituents of *Scutellaria baicalensis* Georgi are altered during fermentation and how these changes influence its pharmacological properties. This review aims to lay a theoretical groundwork for the clinical utilization of *Scutellaria baicalensis* Georgi and the formulation of innovative therapeutic approaches.

## 1 Introduction

A burgeoning body of evidence suggests the potential role of herbal plants and their natural compounds as herbal remedies. Compared to chemical drugs, herbal drugs possess fewer side effects, safer dosages, and the potential for reduced residual toxicity (1). In recent years, the biotransformation of flavonoid compounds in various traditional herbs has been shown to enhance their health-promoting effects. This has made the study of traditional medicinal plants and medicinal and edible homologous substances a topic of significant interest.

Probiotics are a class of active microorganisms beneficial to the host (2). They improve the host’s health by regulating the gut microbiota, promoting digestion, enhancing immunity, and through other mechanisms. Common probiotics include lactic acid bacteria (such as *Lactobacillus* and *Bifidobacterium*) and yeasts (such as *Saccharomyces cerevisiae*).

In recent years, probiotic fermentation technology has garnered widespread attention in the research of medicinal materials (3). Probiotic fermentation can significantly alter the active components in medicinal materials, enhancing their bioavailability and medicinal value.

**Figure 1:**
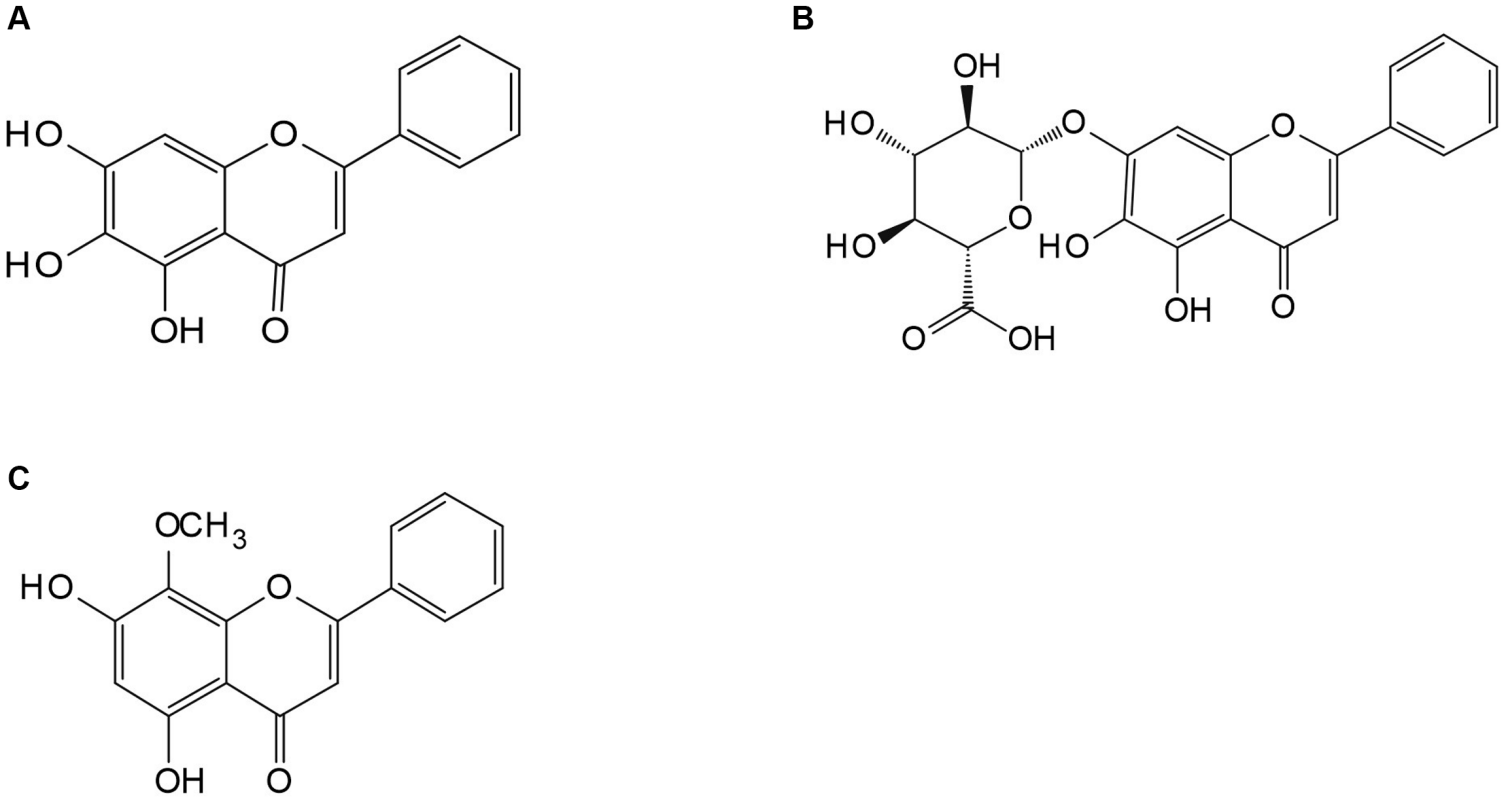
The function of *Scutellaria baicalensis* Georgi.

*Scutellaria baicalensis* Georgi, a species of the *Labiaceae* family, is a medicinal herb widely used in traditional Chinese medicine (TCM). It contains many ingredients, the main active ingredients in *Scutellaria baicalensis* Georgi are flavonoids such as baicalin, baicalein, and wogonin (2). These flavonoids have been studied for their potential beneficial effects on human health, including anti-allergenic, anti-inflammatory, cardiovascular and neuroprotective, hepatoprotective, immunomodulatory, skin barrier-enhancing, and anti-tumor activities (3, 4) (Figure 1). As a result, the Chinese Pharmacopeia has adopted the content of baicalin a flavonoid compound, as a quality control standard for Scutellaria baicalensis Georgi.

Although flavonoids such as baicalein and baicalin play an important role, their content in *Scutellaria baicalensis* Georgi is very low. The content of baicalin is 7.98%, and the content of baicalein ranges from 0.1 to 1.5%. Traditional extraction techniques for *Scutellaria baicalensis* Georgi, include water extraction, alcohol extraction, warm immersion, and decoction methods, each with its own unique advantages and limitations. The water extraction method is favored for its simplicity and cost-effectiveness, making it especially suitable for large-scale production. However, it may not be highly efficient for components with low solubility, and the extended extraction time could potentially degrade heat-sensitive components. The alcohol extraction method has a significant advantage in its high extraction efficiency, which is particularly suitable for extracting flavonoids. Nevertheless, the use of organic solvents not only increases production costs but also poses safety risks and environmental concerns. The warm immersion method is straightforward to operate and is especially suitable for extracting heat-stable components. However, its extraction speed is slower, and it is not suitable for components that are sensitive to heat (5), which contributes to the low extraction efficiency of baicalein and baicalin and thus limits the further application and development of *Scutellaria*.

In recent years, numerous studies have demonstrated that *Lactobacillus* and other probiotic fermentation processes can be used as an alternative method to overcome extraction difficulties. Generally, fermentative techniques have been observed to increase the extraction yield of a variety of plants under relatively mild extraction conditions, such as low temperatures and shorter processing times. For instance, the content of the active ingredient, known as ginsenoside, in *Rhodiola rosea*, has been increased by about 48.45% through the process of microbial fermentation (6). Additionally, some members of *Lactobacillus* and other probiotics are believed to have beneficial effects on host health when ingested. Herbal medicines fermented by *Lactobacillus* and other probiotics may benefit from the strain, increasing the content and activity of their chemical components, and even producing more stimulating metabolites that can be directly absorbed by the stomach and intestines, resulting in the synergistic effect of the various active components produced by fermentation (7, 8).

Therefore, this study will review the pharmacological properties and applications of *Scutellaria baicalensis* Georgi under the condition of probiotic fermentation, providing a reference for further exploration of its medicinal potential.

## 2 The chemical components of *Scutellaria baicalensis* Georgi and extraction methods

**Figure 2:**
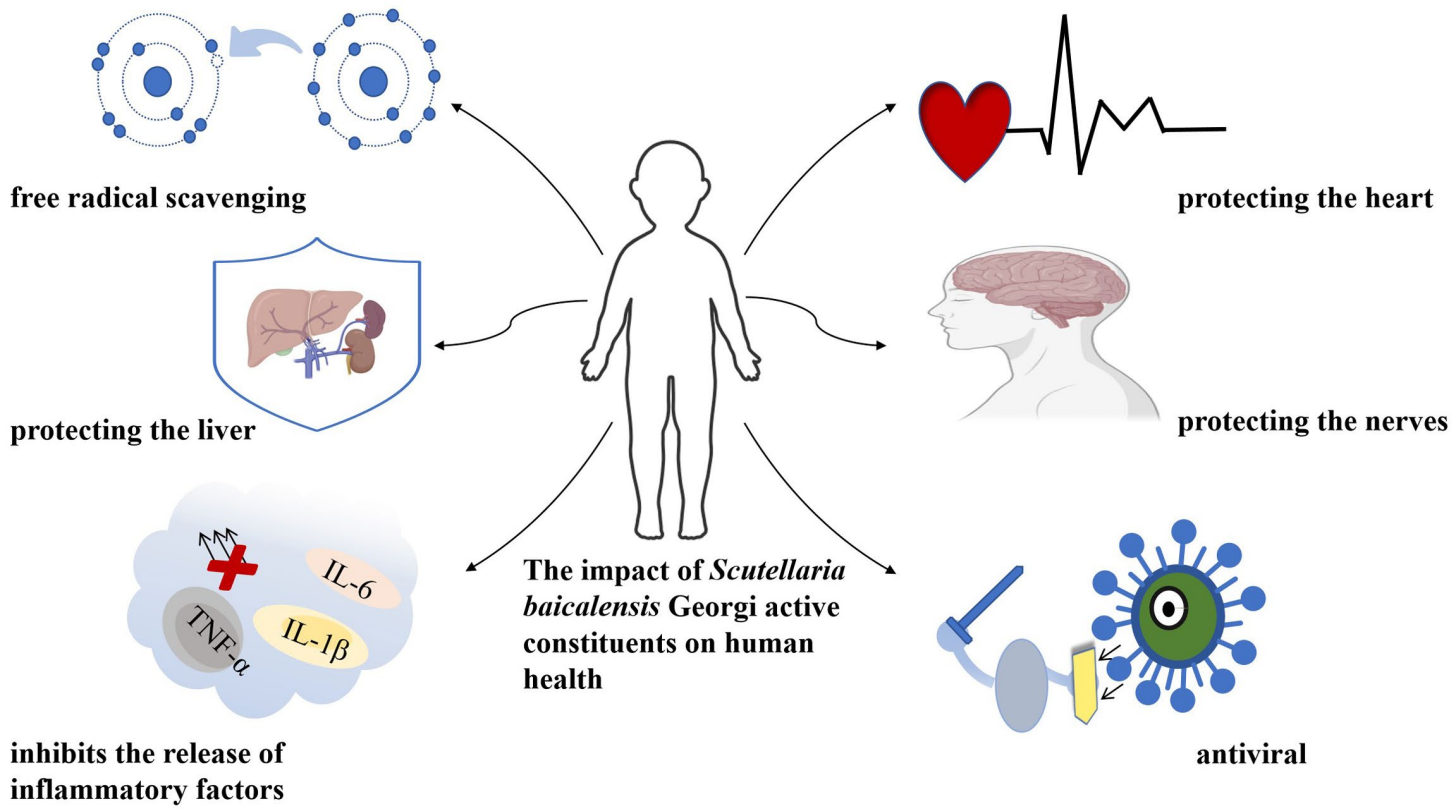
The medicinal plant *Scutellaria baicalensis* Georgi. **(A)** Baicalin; **(B)** baicalein; **(D)** wogonin.

*Scutellaria baicalensis* Georgi (Figure 2), a plant in the *Lamiaceae* family, is documented in China’s Shennong Materia Medica Classic. It has been widely utilized in the clinical treatment within TCM for over 153 various diseases. Since 1973, various chemical components of *Scutellaria baicalensis* Georgi have been extracted using a variety of separation methods. Volatile oil components in *Scutellaria baicalensis* Georgi can be gently and efficiently extracted using organic solvent extraction methods, which may involve either reflux or cold immersion techniques (9). Moreover, high-speed counter-current chromatography (HSCCC) and silica gel column chromatography are two highly efficient chromatographic techniques employed for the separation of flavonoids and their glycosides from the stems and leaves of *Scutellaria baicalensis* Georgi (10, 11). These methods offer a precise means for the purification of active ingredients. To date, more than 40 components have been identified, primarily comprising flavonoids and their glycosides, terpenes, and volatile oils (12) (Table 1). Moreover, the main chemical components of both the aboveground and underground parts of *Scutellaria baicalensis* Georgi are similar, containing a large number of flavonoids, although there are variations in specific compounds. Additionally, small amounts of organic acids, volatile oils, polysaccharides, and inorganic elements are also present. These compounds have antipyretic, anti-inflammatory, antimicrobial, antitumor, and antioxidant properties, and antioxidant properties, and they have demonstrated therapeutic effects on diseases of the digestive, cardiovascular, and nervous systems.

**Table 1:** Components of *Scutellaria baicalensis* Georgi.

Component	Origin	Ref.
Baicalein	Root, stem and leaf	(2)
Baicalin	Root, stem and leaf	(2)
Scutellarin	Root and stem	(9)
Scutellarin-I	Root, stem and leaf	(9)
Skullcapflavone	Root	(13)
Benzyl alcohol	Root	(13)
Lutein	Root	(13)
β-carotene	Root	(13)
β-Sitosterol	Root	(13)
Ligustrin A	Root	(13)
Dihydrobaicalin-7-O-glucoside	Root	(13)

### 2.1 Flavonoids and glycosides

Flavonoid compounds are a type of secondary metabolite that are widely found in plants. They primarily originate from glucose, synthesized through the shikimic acid pathway and acetic acid-malonic acid pathway, which produces hydroxycinnamic acid and three molecules of acetic acid, ultimately leading to the synthesis of chalone (14). Since the late 1970s, over 40 different polyphenols, including various forms of flavonoids such as flavonols, dihydro flavonoids, and chalcones, as well as flavonoids themselves, have been isolated and identified from *Scutellaria baicalensis* Georgi. The most representative components of these polyphenols are baicalin, baicalein, wogonoside, and wogonin (15), which have been extensively studied for their potential beneficial effects.

Baicalin, the predominant flavonoid in the traditional Chinese medicine *Scutellaria baicalensis* Georgi. It is a pale yellow needle-shaped crystal, nearly insoluble in water but readily soluble in alkaline solutions. Experiments have shown that baicalin and baicalein in *Scutellaria baicalensis* Georgi can inhibit the growth of many Gram-positive and negative bacteria. Furthermore, baicalin at varying concentrations has been demonstrated to treat ulcerative colitis to different extents (16), potentially by inhibiting the gene expression of the peripheral blood interleukin-4 receptor (IL4R), interleukin-6 receptor (IL6R), and interleukin-23 receptor (IL-23R). This indicates that the mechanism by which baicalin alleviates symptoms of ulcerative colitis involves the suppression of inflammatory factor secretion, thereby exerting its anti-inflammatory action.

Currently, the extraction methods of total flavonoids from *Scutellaria baicalensis* Georgi include decocting, reflux extraction, ultrasonic extraction, supercritical fluid extraction, microwave-assisted extraction, and semi-bionic extraction. The specific operation of the decoction method involves placing the *Scutellaria baicalensis* Georgi powder into a container (16), first adding an appropriate amount of water to soak it, which facilitates the dissolution of flavonoid substances. Next, after soaking, the *Scutellaria baicalensis* Georgi powder is heated together with the water until it reaches boiling point, and then kept at a gentle simmer for a predetermined period of time. After the decoction is finished, let the mixture cool down slightly, then filter it using filter paper or cloth to collect the filtrate. The collected filtrate is concentrated to reduce the amount of solvent and thus increase the concentration of flavonoid compounds. To remove insoluble materials, it can be filtered again or allowed to stand and precipitate. Finally, the concentrated flavonoid extract is dried using techniques such as freeze-drying or spray drying to obtain a solid extrac. The ultrasonic extraction method involves drying (13) the *Scutellaria baicalensis* Georgi powder and mixing it with ethanol at a specific ratio, followed by placing the mixture into an ultrasonic extraction apparatus for extraction. After the ultrasonic extraction is completed, take out the extract liquid and filter it to remove the insoluble solid residues. Collect the filtered liquid and use techniques such as rotary evaporation or freeze drying to remove the solvent, thereby obtaining the flavonoid extract. Finally, use analytical techniques such as UV–Vis spectrophotometry or High-Performance Liquid Chromatography (HPLC) to determine the content of flavonoids in the extract, to assess the efficiency of the extraction. You et al. (13) employed an ultrasonic extraction method to successfully extract baicalin and baicalein from *Scutellaria baicalensis* Georgi, and conducted a response surface methodology (RSM) test to analyze the effects of the solid–liquid ratio, ethanol concentration, and ultrasonication time on the extraction yields of baicalein and wogonin. And this method offers advantages such as simplicity of operation, cost-effectiveness, and the absence of toxic reagents or environmental pollution, rendering it an ideal choice for flavonoid extraction from *Scutellaria baicalensis* Georgi.

### 2.2 Volatile oil

The volatile oil components identified in *Scutellaria baicalensis* Georgi, are intricate and consist of a diverse array of bioactive substances. These substances possess a multitude of biological activities that surpass those of antioxidant, anti-inflammatory, antimicrobial, and anti-tumor properties. Steam distillation was utilized by Nikbin et al. (17) to extract volatile oil from *Scutellaria baicalensis* Georgi, from which several components were isolated and identified. The specific operation of the steam distillation method is as follows (17): first, the *Scutellaria baicalensis* Georgi material is crushed into coarse powder and then loaded into the distillation flask. Next, a heat source is used to heat the distillation flask, causing the volatile components in the *Scutellaria baicalensis* Georgi material to evaporate along with the steam produced. These steam-borne volatile components pass through the condenser, where they condense into a liquid and then flow into the receiver. Since the liquid contains a mixture of water and volatile oil, and due to their different densities, the volatile oil and water can be separated using a separatory funnel. Finally, the separated volatile oil is subjected to a drying process to remove residual moisture and is stored in a sealed, light-proof container to maintain its stability. The predominant components were trans-carbophyllene (24.8%), big-root geranylene-D (7.9%), linalool (7.4%), α-Caryophyllene (4.9%), patchouliene (4.7%), and menthone (1.1%). These volatile oils possess a pleasant aroma and sweet taste, as well as notable antibacterial effects against certain gram-positive and gram-negative bacteria, including *Bacillus subtilis* and *Klebsiella pneumoniae* (18).

### 2.3 Polysaccharides

he main polysaccharides in *Scutellaria baicalensis* Georgi, known as WSPS-1, WSPS-2, and WSPS-3, are composed of glucose, galactose, and arabinose (19). These polysaccharides have been found to exhibit various beneficial properties, including antioxidant, antiviral, and immunomodulatory activities. Studies have shown that the polysaccharide content in different processed forms of *Scutellaria baicalensis* Georgi varies, with the lowest content at 5% in grey *Scutellaria baicalensis* Georgi and the highest at 12% in fried *Scutellaria baicalensis* Georgi (20). Additionally, the polysaccharide content in *Scutellaria baicalensis* Georgi from different regions or varieties within the same region can be significantly different, yet it is generally maintained between 5.26–13.21%. Furthermore, the polysaccharide content in *Scutellaria baicalensis* Georgi is highly dependent on its source and the processing method used. Cui et al. (21) isolated and purified a polysaccharide, SP1-1, from *Scutellaria baicalensis* Georgi using alcohol immersion extraction and demonstrated that SP1-1 can significantly reduce experimental colitis induced by dextran sulfate (DSS) in mice.

### 2.4 Other chemical components

In addition to the aforementioned chemical constituents, β-sitosterol, benzoic acid and benzyl alcohol have been isolated and identified from *Scutellaria baicalensis* Georgi. Trace elements such as iron, manganese, copper, zinc, nickel, chromium, strontium and others have also been discovered in the plant (22). Despite this, the role of these components in the action of *Scutellaria baicalensis* Georgi is yet to be fully elucidated and warrants further investigation.

## 3 Advantages of traditional Chinese medicine fermented by probiotic

**Figure 3:**
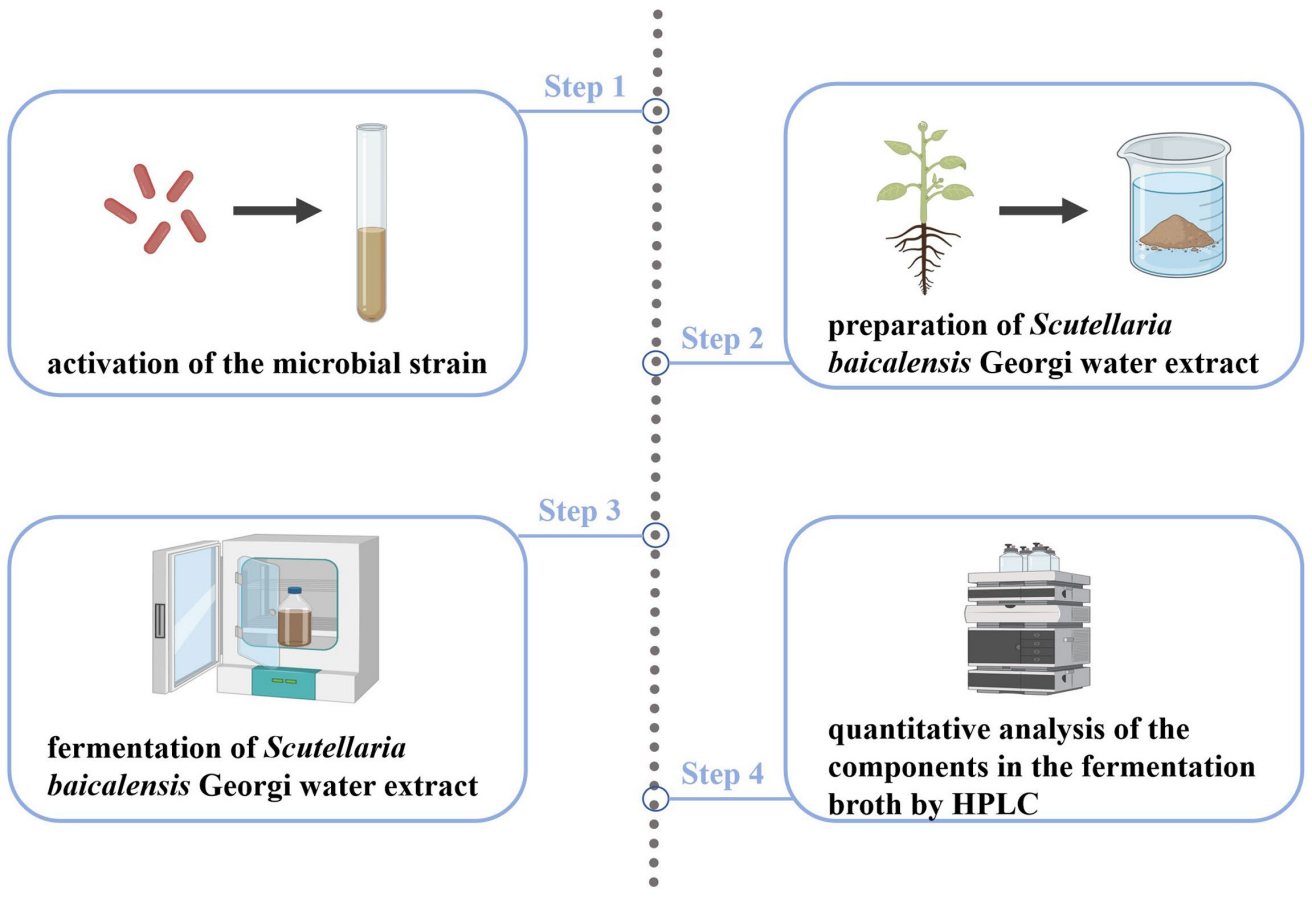
Advantages of traditional Chinese medicine fermented by probiotic.

In recent years, the rapid development of microbial fermentation technology and in-depth research into the modernization of traditional Chinese medicine has led to microbial fermentation and transformation of traditional Chinese medicine gaining widespread attention and becoming a new way to produce new active compounds with medicinal value (23). The fermentation conditions are generally at normal temperature and pressure, which can reduce the damage to the active ingredients in traditional Chinese medicine, improve efficacy, and change the properties (24). Moreover, the components of traditional Chinese medicine can also influence the metabolites of microorganisms, forming new components that are easily absorbed (25). Additionally, microbial interaction with traditional Chinese medicine can reduce toxic components present in traditional Chinese medicine (26) (Figure 3). The exploration and research of microbial fermentation in modern traditional Chinese medicine involve the following aspects.

**Figure 4:**
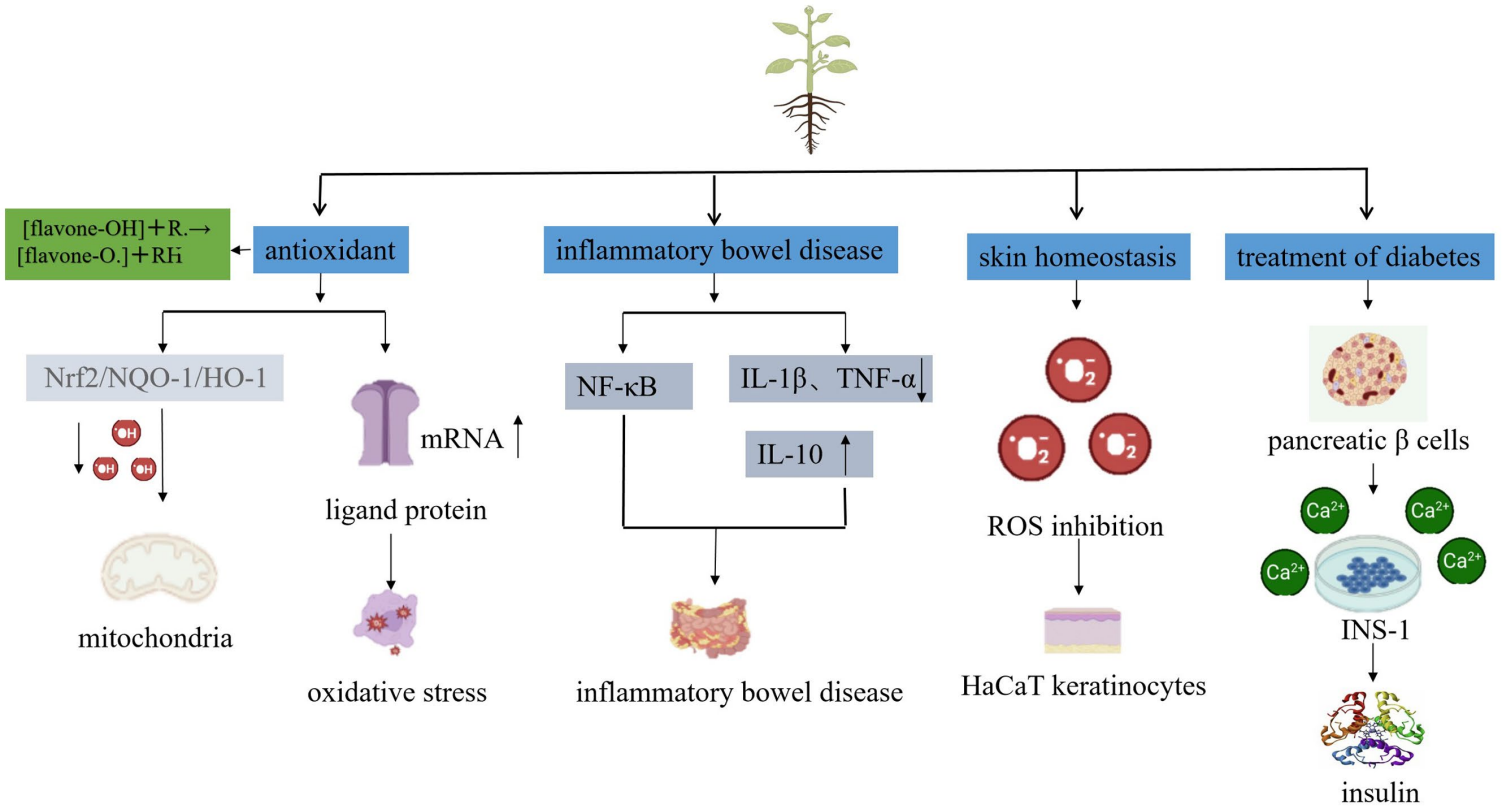
Baicalin transformation structure.

1. Probiotics have been demonstrated to produce cellulase, cellobiase, protease, galactose, and carbohydrate-active enzymes in metabolism, which can break down the cell wall of medicinal plants, reduce the encapsulation of polysaccharides and other active ingredients, and release more alkaloids, flavonoids, glycosides, organic acids, terpenes, and other active ingredients. This can result in improved efficacy (27). For instance, a study found that the mass fraction of total flavonoids, saponins, alkaloids, crude polysaccharides, and total polysaccharides in Leonurus heterophyllous increased by 55.14, 49.21, 127.28, 55.42, and 57.31%, respectively, after the compound fermentation of *Candida utilis*, *Lactobacillus casei*, and *Enterococcus faecalis* (28). Other studies showed that the saponin content of red ginseng nearly tripled after microbial fermentation (29). Baicalin and wogonin can be directly absorbed in the small intestine by transforming into baicalein (Figure 4) and other aglycones, which can play a pharmacological role, even though the content of baicalein and wogonin is usually between 0.10 and 1.60%, and 0.01 and 0.30%, respectively (30). However, a natto fermentation of baicalin has been observed to have a conversion rate of 96.87% for baicalin and 86.38% for wogonin (31).
2. Some Chinese herbal medicines have been found to have a toxicity that can cause damage to the body. However, probiotic fermentation can be used to significantly reduce this toxicity. For instance, the toxicity of Annona fruit has been reduced after fermentation with ganoderma lucidum, mushroom, schizophyllum, and other fungi (32). Additionally, the toxicity of *Strychnos nux-vomica* has been reduced after being fermented by six kinds of fungi, such as *Schizophyllum* commune Fr., *Shiraia bambusicola* P. Henn., and *Coriolus versicolor* (L. ex Fr.) Quel., while still maintaining its original analgesic and anti-inflammatory effects (33). This fermentation process not only reduces toxicity but also preserves the therapeutic properties of the herbs, which is essential for the development of safer pharmaceutical products.
3. The production of new active substances can be achieved through the fermentation of rhubarb, senna leaves, Panax notoginseng rhizomes, and *Scutellaria baicalensis* Georgi using probiotics, such as *Bacillus subtilis*, or medicinal fungi, such as Ganoderma lucidum. For instance, intestinal flora can metabolically transform the aloin and senna present in rhubarb and senna leaves into their corresponding aglycones, thereby playing a pharmacological role (34). Furthermore, fermentation of Panax notoginseng rhizomes with probiotics has resulted in the production of a new component, ginsenoside Rh_4_ (35). Similarly, a bidirectional solid fermentation of *Scutellaria baicalensis* Georgi using the medicinal fungus Ganoderma lucidum has enabled the isolation of 6-o-β-D-glucose-cyclo astragalus alcohol from the product (36).
4. *Lactobacillus plantarum* and *Lactobacillus rhamnosus* (37) have been shown to be effective in increasing the weight of piglets, reducing the diarrhea rate, improving immunity, and regulating the intestinal microbiota of piglets when used as a feed additive made from fermented *Scutellaria baicalensis* Georgi residue (38). Further, Yang et al. (39) demonstrated that the product obtained from the fermentation of *Scutellaria baicalensis* Georgi dregs with *Trichoderma echinococcus* had strong antibacterial activity against *Proteus*, *Salmonella, Bacillus subtilis*, *Staphylococcus aureus*, and *Escherichia coli*, with an inhibition zone diameter of 19.1–29.9 mm, but no antibacterial effect was observed with the *Scutellaria baicalensis* Georgi dregs and *Trichoderma echinococcus* mixture.

However, probiotic fermentation has limitations, including low fermentation efficiency, difficulty in controlling the fermentation process, and sensitivity to fermentation conditions (40). To improve these limitations, modern fermentation techniques such as liquid and solid fermentation, and bidirectional fermentation with medicinal fungi can be used. It is also possible to optimize pH, temperature and incubation time, which can increase the yield of active natural products. To screen for probiotics with higher yields and functionality, microbiomics and synthetic biology techniques can be utilized. Microbiomics techniques can be used to analyze and screen suitable probiotics for herbal fermentation (41), while synthetic biology can be used to improve hydrolase activity and other properties through protein engineering and metabolic engineering to provide more effective enzymes or microorganisms for herbal fermentation. Through these approaches, synthetic microbial communities can be designed and constructed, and the fermentation process can be optimized to achieve high yields of bioactive ingredients.

## 4 Effect and mechanism of *Scutellaria baicalensis* Georgi fermented by probiotic

### 4.1 Antioxidant and free radical scavenging activities

Baicalin can increase the activities of Superoxide dismutase (SOD) and Glutathione (GSH) in the hippocampal tissue of aging rats, which can remove oxygen free radicals, protect the body from free radical damage (42), and reduce the concentration of MDA. Additionally, by down-regulating the expression level of Caspase-3, apoptosis is inhibited, thus alleviating cell damage.

The microbial fermentation of *Scutellaria baicalensis* Georgi generates specific enzymes that can convert baicalein and baicalin into more bioavailable forms, potentially enhancing their antioxidant activities. Additionally, organic acids produced during fermentation interact with baicalein and baicalin to form complexes, which increase their solubility in water and, consequently, their bioavailability and antioxidant efficacy within the body (43). The capacity of baicalein and baicalin to scavenge hydroxyl radicals is correlated with their concentration; the fermentation process may bolster the antioxidant effects of these compounds by improving their interaction with and neutralization of free radicals Baicalin, by relying on its hydroxyl group, is more powerful than the known antioxidant quercetin (44) and has been shown to possess the ability to relieve oxidative stress damage in rat cerebral cortex. It is difficult, however, for baicalin to be directly absorbed in the human body. Once it enters the body, it is metabolized in the liver and enzymatically hydrolyzed or transformed by intestinal flora into baicalein, which increases cell permeability and enters the blood to better play its drug effect (45). In order to enhance traditional processing techniques such as those used in Chinese herbal medicine and decocting, Jeong and Kim (46) has screened a mold that specializes in the fermentation and transformation of baicalin. This process produces baicalein, which has precise efficacy and high bioavailability due to the β-glucuronidase produced by the fungus. This not only improves traditional processing techniques but also increases baicalin bioavailability, thus producing antioxidant, anti-inflammatory, and other effects.

### 4.2 Treatment of inflammatory bowel disease

When the immune system is exposed to external stimuli, it can be destroyed, resulting in an imbalance in the intestinal flora and ultimately leading to the occurrence of Inflammatory Bowel Disease (IBD). The two main types of IBD are Crohn’s Disease (CD) and Ulcerative Colitis (UC). Baicalin has been found to downregulate the levels of pro-inflammatory factors such as IL-1β and TNF-α, as well as upregulate the level of interleukin-10 (IL-10) to inhibit the inflammatory response in rats with UC (47). Additionally, baicalin can aid in mucosal repair and maintain normal intestinal mucosal immunoregulation. The Nuclear Factor-Kappa B (NF-κB) signaling pathway is a classic signaling pathway involved in ulcerative colitis. The NF-κB transcription factor family plays a crucial role in cell differentiation, proliferation, apoptosis, inflammation, and stress response. Studies have shown that baicalein can interfere with NF-κB signal transduction, thus exhibiting anti-inflammatory properties (48). Additionally, the PTEN-induced putative kinase 1 (PINK1)/E3 ubiquitin ligase (Parkin) signal pathway is a well-known mitochondrial autophagy pathway. Extracts from *Scutellaria baicalensis* Georgi have been found to activate the PINK1/Parkin signal pathway, thus increasing the mitochondrial autophagy level of the colon (49). Furthermore, it has been demonstrated that *Scutellaria baicalensis* Georgi extracts can inhibit the activation of the NLRP3 inflammatory body as well as reduce the levels of myeloperoxidase (MPO), lipid peroxide (LPO), interleukin-1β (IL-1β), interleukin-18 (IL-18) and tumor necrosis factor-α (TNF-α), thereby reducing the inflammatory response. Fermented baicalein positively affects inflammatory bowel disease (IBD) through several mechanisms. It modulates the function of intestinal immune cells, particularly by affecting the activity of T- and B-cells to improve the immune-mediated processes in IBD (50). In addition, the fermentation process promotes the balance of intestinal flora, utilizing probiotics to improve the intestinal microenvironment of IBD patients (48). The metabolites produced by probiotics during fermentation may have additional anti-inflammatory or immunomodulatory effects, providing a new mechanism for baicalein treatment of IBD. Also, fermentation may enhance the therapeutic effect by changing the structure of baicalin to improve its solubility and absorption in the gut. These combined effects suggest that fermented baicalin has multifaceted potential in the treatment of IBD, but further clinical studies and experiments are needed to verify these potential effects. Seok et al. (51) conducted an *in vitro* bacteriostasis test, polysaccharide content determination, and animal test on the fermented *Scutellaria baicalensis* Georgi, which was produced by *Lactobacillus rhamnosus*, and found that the effective components of *Scutellaria baicalensis* Georgi were released in high quantities after fermentation. Additionally, *Lactobacillus rhamnosus* has the effect of regulating the gastrointestinal flora and has a synergistic effect with *Scutellaria baicalensis* Georgi, which not only has the ability to treat piglet colibacillosis, but also improves its immunity and growth performance. Compared to traditional Chinese medicine and enrofloxacin treatment, the effectiveness of this treatment was better, resulting in improved production performance in the later stages of treatment.

### 4.3 Improvement of skin barrier

Atopic Dermatitis (AD) is a chronic, recurrent eczematous skin condition with clinical manifestations of dry skin and intense itching (52). Its development is complex and involves various genetic, immune, environmental, and other causes and diseases (53, 54). Additionally, the growth and differentiation of keratinocytes and the reduction of larger dermal connective tissue may also contribute to AD and other age-related skin problems. With increased standards of living, people are more conscious of skin health and care, and prefer natural cosmetics resources derived from medicinal plants that are safer than chemicals, with fewer side effects (55). *Scutellaria baicalensis* Georgi is a medicinal plant containing a significant amount of flavonoids such as baicalin and baicalein. Flavonoids are known as powerful natural antioxidants, and may play an important role in preventing skin barrier dysfunction (56). Studies have shown that a Scutellaria extract, after fermentation by *Lactobacillus plantarum*, can prevent human skin barrier dysfunction, eventually helping to relieve AD. The total polyphenol content of the FE extract was 35.9 mg/g, which was higher than that of the Water Extraction (WE) and 70% Ethanol Extraction (EE) at 29.84 mg/g and 23.59 mg/g, respectively. After lactic acid fermentation, the content of polyphenols was 40% higher than that of the water extraction (57). Zhou et al. (58) identified a strain of Penicillium decumbens f3-1 that could ferment the water extract of *Scutellaria baicalensis* Georgi, converting the baicalin to wogonin, with a conversion rate of 91%. Furthermore, it was found that the water extract of *Scutellaria baicalensis* Georgi after fermentation was more effective than before fermentation in inhibiting interleukin-1 beta (IL-1 β) and interleukin-8 (IL-8) induced by *Propionibacterium acnes* (*P. acnes*). Thus, fermentation was shown to significantly improve the anti-inflammatory and acne-removing activity of the *Scutellaria baicalensis* Georgi water extract.

### 4.4 Treatment of type 2 diabetes

Diabetes mellitus (DM) is one of the most widespread diseases in the world, and it can be divided into type 1 diabetes, an autoimmune disorder associated with a complete lack of insulin secretion, and type 2 diabetes, which is a result of pancreatic islet β cell failure or insulin resistance. Both types lead to hyperglycemia, excessive urination, blurred vision, changes in body mass, compensatory thirst, lethargy, and alterations in energy metabolism, with type 2 diabetes being the more common of the two. While insulin injections and the use of metformin can help to reduce the symptoms and complications of diabetes, as well as maintain blood glucose stability, clinical studies have found that these treatments can cause various side effects, such as gastrointestinal reactions, hypoglycemia, and insulin resistance (59), which can further damage the pancreatic islet cells of the patient.

Research has found that baicalin can inhibit the Kv channel of pancreatic islet β cells, increasing intracellular calcium concentration, reducing the inflammatory damage of INS-1 cells and promoting insulin secretion (60). Xu et al. (61) further demonstrated that *Lactobacillus brevis* RO1 β- Glucuronidase can promote the transformation of baicalin into baicalein and wogonin into wogonin during fermentation, thereby improving diabetes by regulating intestinal flora. Wang Tao also studied the microbial transformation of traditional Chinese medicine *Scutellaria baicalensis* Georgi and used natto bacteria to ferment it. Through thin-layer chromatography and high-performance liquid chromatography, it was verified that the enzymes produced during the fermentation process could reduce the content of flavonoid glycosides in *Scutellaria baicalensis* Georgi by β- Hydrolysis of the glucuronic acid bond, efficiently transforming baicalin and baicalin and thus reducing blood sugar levels (62).

### 4.5 Effect of drug metabolism on the components of *Scutellaria baicalensis* Georgi

The effect of drug metabolism on the components of baicalein is mainly reflected in the biotransformation of baicalin, a process that can produce metabolites with different biological activities. *In vivo*, baicalin is metabolized to produce active components such as baicalein and baicalein (63), which may contribute to antioxidant, anti-inflammatory and other pharmacological effects. In addition, drug metabolism may affect the bioavailability and efficacy of baicalin constituents, which may have an important impact on the clinical application of *Scutellaria baicalensis* Georgi.

## 5 Summary and prospect

*Scutellaria baicalensis* Georgi has a long history of medicinal application in China. As a widely utilized herbal medicine, *Scutellaria baicalensis* Georgi has demonstrated properties such as antioxidant, anti-inflammatory, and anti-tumor activities, as well as anti-allergenic effects. The fermentation of this herb with probiotics and other microorganisms can lead to an increase in the concentration of compounds like baicalin, potentially enhancing its therapeutic efficacy. Nevertheless, further research is necessary to optimize the application of *Scutellaria baicalensis* Georgi in medical practice.

The study of drug metabolism has revealed that baicalein is the key active compound in *Scutellaria baicalensis* Georgi. In addition to improving the dissolution rate of effective ingredients, microbial fermentation of this plant also focuses on the transformation of baicalin, its natural drug precursor, to baicalein via structural changes, thus simulating the metabolic pathway of *Scutellaria baicalensis* Georgi in the human body and producing a variety of metabolites. Therefore, it is beneficial to select suitable probiotics to enhance the pharmacological effect of *Scutellaria baicalensis* Georgi. Additionally, the extraction and purification of effective ingredients such as baicalein is an important factor that constrains its clinical use. While traditional decoction and processing methods are no longer sufficient to support its rapid development, modern technologies such as enzymolysis combined with ultrasound extraction, macroporous resin for separation and purification, are gaining traction. These methods can help improve the utilization rate of *Scutellaria baicalensis* Georgi.

In addition to baicalin and baicalein, many other effective components of *Scutellaria baicalensis* Georgi have yet to be developed into pharmaceuticals. Moreover, the majority of research on *Scutellaria baicalensis* Georgi has focused on the root, while its stem, leaf, and fruit have been largely neglected. It is therefore necessary to increase the utilization of the aboveground parts of *Scutellaria baicalensis* Georgi by studying the chemical components and pharmacological effects of these other parts. Exploring their characteristics and active ingredients is essential in order to meet the needs of clinical practice.

Currently, there is a great deal of research on the pharmacological actions and mechanisms of the active ingredients and extracts of *Scutellaria baicalensis* Georgi. New pharmacological effects are being discovered continuously, yet comprehensive and systematic research is lacking. To address this, molecular and cellular-level research should be conducted. Additionally, there are few studies on the pharmacokinetics and toxicology of this plant, so further toxicity tests *in vivo* and *in vitro* should be carried out to provide an experimental basis for the rational use of this plant in clinical settings.

## Author contributions

FG: Writing – original draft. CL: Writing – review & editing. JD: Conceptualization, Writing – review & editing. JL: Investigation, Writing – review & editing. ZC: Data curation, Writing – review & editing. ZX: Formal analysis, Writing – review & editing. TW: Writing – review & editing.
